# Preconditioning of the motor network with repetitive navigated transcranial magnetic stimulation (rnTMS) to improve oncological and functional outcome in brain tumor surgery: a study protocol for a randomized, sham-controlled, triple-blind clinical trial

**DOI:** 10.1186/s13063-023-07640-2

**Published:** 2023-10-04

**Authors:** Melina Engelhardt, Ulrike Grittner, Sandro Krieg, Thomas Picht

**Affiliations:** 1 Department of Neurosurgery, Charité - Universitätsmedizin, corporate member of Freie Universität Berlin and Humboldt-Universität zu Berlin, Charitéplatz 1, Berlin, 10117 Germany; 2 Einstein Center for Neurosciences, Charité - Universitätsmedizin, corporate member of Freie Universität Berlin and Humboldt-Universität zu Berlin, Charitéplatz 1, Berlin, 10117 Germany; 3 International Graduate Program Medical Neurosciences, Charité - Universitätsmedizin, corporate member of Freie Universität Berlin and Humboldt-Universität zu Berlin, Charitéplatz 1, Berlin, 10117 Germany; 4 Institute of Biometry and Clinical Epidemiology, Charité - Universitätsmedizin, corporate member of Freie Universität Berlin and Humboldt-Universität zu Berlin, Charitéplatz 1, Berlin, 10117 Germany; 5 Department of Neurosurgery, Universitätsklinikum Heidelberg, Ruprecht-Karls Universität Heidelberg, Heidelberg, 69120 Germany; 6 Cluster of Excellence Matters of Activity, Image Space Material, Humboldt-Universität zu Berlin, Unter den Linden 6, Berlin, 10099 Germany

**Keywords:** Glioma, Resection, Transcranial magnetic stimulation (TMS), Prehabilitation, Preconditioning, Motor, Randomized controlled trial

## Abstract

**Background:**

The extent of resection of glioma is one of the most important predictors of the survival duration of patients after surgery. The presence of eloquent areas within or near a tumor often limits resection, as resection of these areas would result in functional loss and reduced quality of life. The aim of this randomized, triple-blind, sham-controlled study is to investigate the capability of repetitive navigated transcranial magnetic stimulation (rnTMS) over the primary motor cortex to facilitate the functional reorganization of the motor network.

**Methods:**

One hundred forty-eight patients with tumors in movement-relevant areas will be included in this randomized, sham-controlled, bicentric, triple-blind clinical trial. Patients considered at high risk for postoperative motor deficits according to an initial nTMS assessment will receive inhibitory rnTMS at 1 Hz for 30 min followed by a short motor training of 10 min. Stimulation will be applied to the fiber endings of the corticospinal tract closest to the tumor based on individualized tractography. Stimulation will be performed twice daily for each 30 min for 5–28 days depending on the individually available time between study inclusion and surgery. The intervention is controlled by a sham stimulation group (1:1 randomization), where a plastic adapter will be placed on the coil. We expect a comparable or better motor status 3 months postoperatively as measured by the British Medical Research Council (BMRC) score for the affected upper extremity (non-inferiority) and a higher rate of gross total resections (superiority) in the rnTMS compared to the sham group.

**Discussion:**

The generated reorganization of the brain’s areas for motor function should allow a more extensive and safer removal of the tumor while preserving neurological and motor function. This would improve both survival and quality of life of our patients.

**Trial registration:**

DRKS.de DRKS00017232. Registered on 28 January 2020.

**Supplementary Information:**

The online version contains supplementary material available at 10.1186/s13063-023-07640-2.

## Administrative information

Note: the numbers in curly brackets in this protocol refer to SPIRIT checklist item numbers. The order of the items has been modified to group similar items (see http://www.equator-network.org/reporting-guidelines/spirit-2013-statement-defining-standard-protocol-items-for-clinical-trials/).

**Table:** 

Title {1}	Preconditioning of the motor network with repetitive navigated transcranial magnetic stimulation (rnTMS) to improve oncological and functional outcome in brain tumor surgery: a study protocol for a randomized, sham-controlled, triple-blind clinical trial
Trial registration {2a and 2b}.	The trial was registered at DRKS.de (DRKS00017232) on 28.01.2020.
Protocol version {3}	Version 1, 12.06.2023
Funding {4}	This clinical trial is funded by the Federal Ministry of Education and Research (Bundesministerium für Bildung und Forschung (BMBF), Deutsches Zentrum für Luft- und Raumfahrt (DLR) Projektträger Gesundheit) by grant number 01KG2302.
Author details {5a}	Melina Engelhardt: Charité - Universitätsmedizin, corporate member of Freie Universität Berlin and Humboldt-Universität zu Berlin, Department of Neurosurgery, Charitéplatz 1, 10117 Berlin, Germany Melina Engelhardt: Charité - Universitätsmedizin, corporate member of Freie Universität Berlin and Humboldt-Universität zu Berlin, Einstein Center for Neurosciences, Charitéplatz 1, 10117 Berlin, Germany Melina Engelhardt: Charité - Universitätsmedizin, corporate member of Freie Universität Berlin and Humboldt-Universität zu Berlin, International Graduate Program Medical Neurosciences, Charitéplatz 1, 10117 Berlin, Germany Ulrike Grittner: Charité - Universitätsmedizin, corporate member of Freie Universität Berlin and Humboldt-Universität zu Berlin, Institute of Biometry and Clinical Epidemiology, Charitéplatz 1, 10117 Berlin, Germany Sandro Krieg: Department of Neurosurgery, Klinikum rechts der Isar, Technische Universität München, Munich, Germany Thomas Picht: Charité - Universitätsmedizin, corporate member of Freie Universität Berlin and Humboldt-Universität zu Berlin, Department of Neurosurgery, Charitéplatz 1, 10117 Berlin, Germany
	Thomas Picht: Cluster of Excellence Matters of Activity. Image Space Material, Humboldt-Universität zu Berlin, Unter den Linden 6, 10099 Berlin, Germany
Name and contact information for the trial sponsor {5b}	Charité – Universitätsmedizin Berlin, Charitéplatz 1, 10117 Berlin, Germany
Role of sponsor {5c}	The funding source {4} and sponsor {5b} had no role in the design of this study and will not have any role during its execution, analyses, interpretation of the data, or decision to submit results.

## Introduction

### Background and rationale {6a}

Around 8300 cancers of the central nervous system and 6140 deaths are reported in Germany every year [1]. These cancers are associated with 150,993 disability-adjusted life years reported in 2016 [1]. Furthermore, an increase in sick leave is observed in these patients before surgery with a return-to-work rate of only 50% 1 year after surgery [2], thus presenting a major socioeconomic burden. While maximal surgical resection has been linked with improved overall survival, up to 50% of intrinsic brain tumors are not completely resected due to infiltration of functional brain tissue. In addition, up to 25% of patients with brain tumors in motor areas suffer from new or worsened motor deficits postoperatively. Both, incomplete tumor resection and impaired neurological status, are directly correlated with decreased life expectancy [3, 4]. While surgical resection is the most effective treatment option for intrinsic brain tumors, traditionally, tumors that have been demonstrated to infiltrate functional tissue will be diagnosed as inoperable and will be treated conservatively from then on. However, in recent years the concept of neurological functions being confined to specific areas of the brain has been questioned and a novel concept of function being represented in dynamic cortico-subcortical networks has been introduced [5]. This paradigm shift also implies that function can move, for example, due to the presence of a pathology — yet typically, the reshaping is too slow or ineffective to sustainably affect the clinical course of the patient.

A way to increase the extent of resection and improve prognosis would be to promote functional reorganization, where brain areas distant from the tumor take over behavioral functions of eloquent areas at risk. Several case studies have shown proof-of-concept that (non)invasive cortical stimulation can facilitate plastic reorganization in brain tumor patients leading to higher resection rates and improved functional outcomes [6–8]. The most recent of these studies [7] applied direct cortical stimulation to tumor-invaded eloquent brain areas via implanted grid electrodes for approximately 16 days after a partial tumor resection in five patients. They were able to induce functional reorganization in all cases, thus enabling a second surgery with the removal of the former eloquent brain areas without permanent functional deficits. However, many patients suffered from severe side effects such as cerebral seizures. A case study examining a patient with a tumor in Broca’s area applied theta burst stimulation over 12 consecutive days using rTMS to induce plasticity in language areas and observed no severe side effects [8]. However, changes in functional connectivity were only visible in magnetoencephalography measurements but did not influence the extent of resection.

Recent studies investigate more intense stimulation protocols, applying rTMS multiple times per day [9]. These studies showed that days of treatment can be reduced significantly, while reaching similar or higher efficacy. Furthermore, a recent pilot study found no increase in side effects in an accelerated low-frequency repetitive neuronavigated TMS (rnTMS) protocol compared to conventional once-daily rnTMS [10]. A recent clinical trial [11] provided the first evidence for rnTMS to facilitate recovery of postoperative motor deficits in brain tumor patients. Here, already, a stimulation duration of 7 days led to a significantly better motor function compared to the sham group with a number needed to treat of only 2.19. A following clinical trial [12] replicated these results with slightly lower effect sizes. No previous study has investigated the here proposed preconditioning approach of preoperative rnTMS on postoperative clinical and neurooncological outcomes so far.

### Objectives {7}

#### Primary hypotheses

We hypothesize that preoperative rnTMS can induce a functional reorganization of the stimulated motor network, leading to (a) a comparable or better motor status (British Medical Research Council (BMRC) score for upper extremities) 3 months postoperatively (T3) (non-inferiority), and (b) a higher gross total resection rate (superiority) in the rnTMS group compared to the sham group.

#### Secondary hypotheses

The induced functional reorganization will be visible in stronger changes of functional connectivity from baseline to the time point after the intervention (T1) in the intervention group compared to the sham group. Furthermore, rTMS treatment will lead to better health-related quality of life (European Organization for Research and Treatment of Cancer Quality of Life Questionnaire Brain Tumor Module (EORTC QLQ BN20)) and motor function (Action Research Arm Test (ARAT)) compared to the control group at 7 days (T2) and 3 months postoperatively (T3).

### Trial design {8}

**Fig. 1:**
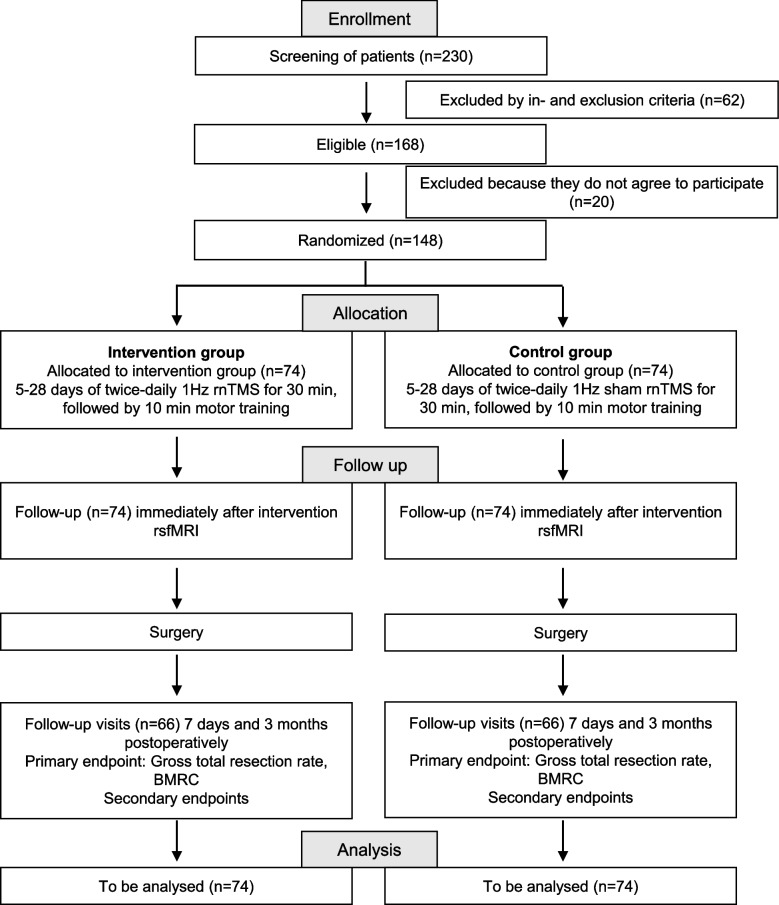
The diagram summarizes the trial intervention scheme and trial flow. Patients are screened for inclusion and exclusion criteria (see {10}). Participation in the study is offered to all eligible patients and patients agreeing to participate will be randomized (see {16}). Equal numbers of patients will be allocated to an intervention and sham-controlled group (see {6b, 11a}). After the intervention, patients will complete one follow-up before surgery as well as two follow-ups postoperatively (see {12, 13}). All randomized patients will be included in the analysis in an ITT framework (see {20})

The trial is designed as a randomized, triple-blind, sham-controlled, bicentric parallel-group two-arm non-inferiority trial. Patients will be allocated 1:1 to the two groups. Patients, outcome assessors, and the trial statistician will be blinded to the group allocation. An overview of the trial timeline can be found in Fig. 1.

## Methods: participants, interventions, and outcomes

### Study setting {9}

All study procedures and rnTMS sessions will be performed at the Charité Berlin and Heidelberg University Hospital. Patients will be recruited from the regular incoming patients. Figure 1 gives an overview of the trial design and patient flow. A current list of participating study sites can be obtained from the coordinating investigator and on the trial register (DRKS00017232), if additional study sites will be included during the course of the study (for example to increase recruitment numbers).

### Eligibility criteria {10}

Subject inclusion criteria

- Suspected supratentorial glioma grade II-IV in a motor-relevant area based on preoperative (Magnetic Resonance Imaging (MRI)
- Infiltration of primary motor cortex or distance between tumor and corticospinal tract below 8 mm [13] based on results of preoperative nTMS assessment and following tractography of the corticospinal tract
- Study participation does not delay surgery
- Age ≥ 18 years at the time of signing the informed consent
- Ability to give written informed consent subject exclusion criteria
- Contraindications for receiving an MRI as assessed by the attending neuroradiologist
- Contraindications for undergoing a TMS examination as assessed by the attending neurosurgeon
- Surgery scheduled within < 5 days after screening for inclusion criteria
- More than one seizure per week based on patient reports or medical records
- Pregnancy
- Missing informed consent

### Who will take informed consent? {26a}

Incoming patients will be screened for eligibility by a study physician during ambulatory care visits or admission to the neurosurgical ward. Eligible patients will be informed about the study procedures and provided with a written study information and informed consent form. Potential study patients will be given at least 24 h between study information and the provision of consent. Consent forms contain a declaration of data privacy and protection alongside contact information in case of questions or complaints. Consent will be obtained in written form and has to be signed by the patient and study physician.

### Additional consent provisions for collection and use of participant data and biological specimens {26b}

The study consent form includes a series of opt-in questions on data reuse and sharing that will not affect participation in the trial itself. Patients can choose to agree to these questions or reject them individually. No biological specimens will be collected.

## Interventions

### Explanation for the choice of comparators {6b}

Participants in the control group will receive sham stimulation over the affected motor cortex followed by a short motor training. The sham rnTMS protocol in this study uses a custom-made plastic adapter placed on the stimulation coil, avoiding the induction of a meaningful electric field at the cortical level. This sham setting elicits a sound comparable to real rnTMS as it allows stimulation at the same intensity as during the real rnTMS intervention.

### Intervention description {11a}

Participants in the intervention arm will receive rnTMS over the affected motor cortex using a Nexstim NBS 5 stimulator with neuronavigation (Nexstim, Helsinki, Finland) followed by a short motor training. The stimulation target is selected by combining TMS with tractography. Functional fibers of the corticospinal tract closest to the tumor and hence, most endangered by resection of the tumor, are identified. RnTMS will be centered over the cortical endings of those fibers. Stimulation is applied at 1 Hz [14] and an intensity of 110% [10]. The duration of the intervention is 30 min per session twice daily (90 min apart) over multiple days to maximize the treatment dose in the short preoperative time [9]. The individual study duration depends on the time available before surgery (≥ 5 days, maximum 28 days). While we assume that longer trial durations will maximize the effects and clinical impact of the stimulation [8, 15], the effects of rnTMS have already been shown after a single session [16]. A short motor training of 10 min will be applied after rnTMS stimulation to facilitate the recruitment of additional networks for motor function while motor fibers in danger during surgery are temporarily inhibited [7]. A set of exercises has been established together with physiotherapists, allowing adaption of the training based on participants’ abilities. See also {13} for a detailed description of the participant timeline and procedures performed at each timepoint.

### Criteria for discontinuing or modifying allocated interventions {11b}

Patients can withdraw from the trial on their own wish at any point without any disadvantages. They will also be released from the trial in case of immediate need for surgery, for example, due to a progressing tumor, or conflicting recommended or necessary medical treatments. Occurrence of serious moderate, serious or severe, life-threatening, or fatal adverse events will lead to exclusion of the participant. In case of moderate, but not serious adverse events treatment may be continued, modified, or discontinued based on the judgment of the independent Data and Safety Monitoring Board (DSMB).

### Strategies to improve adherence to interventions {11c}

To promote participant retention, travel to the hospital can be organized by the investigators and financially compensated (e.g. costs for a taxi). While the beneficial effects of rTMS remain to be demonstrated, we expect a higher degree of resection with the preservation of motor function in the active rTMS arm. In addition, a closer patient connection through daily study participation can positively influence patients’ fear of surgery and mood. The flexible treatment duration tailors to individual patient’s needs and changes in treatment schedules without affecting the completeness of study parameters. Therefore, we expect a high level of compliance on the patients’ side.

### Relevant concomitant care permitted or prohibited during the trial {11d}

No restrictions apply with respect to patients’ medication before or during the trial and treatments before the trial. During the trial, treatment with additional therapeutic brain stimulation methods is not permitted as this would impact the outcome assessment.

### Provisions for post-trial care {30}

No special treatment is planned for patients completing the trial. Patients will receive their treatments and clinical follow-ups according to the standard of care as determined by the treating neurosurgeon. Any patients suffering harm from trial participation will receive the standard treatment for the symptoms they have been experiencing by the medical staff of the respective hospital.

### Outcomes {12}

#### Primary endpoints

The BMRC score for upper extremities will be measured 3 months postoperatively. The BMRC score is scored on a 5-point scale, where 0 corresponds to no muscle contraction in assessed muscles and 5 to normal muscle strength [17]. It has been used in various nTMS studies in brain tumor patients to quantify motor impairment [13, 18]. Furthermore, preservation of function is one of the most important factors influencing quality of life after surgery.

The gross total resection rate (GTR) is additionally measured 7 days postoperatively as one of the most important prognostic markers for the survival of patients. A lower residual tumor volume directly correlates with an improved survival of a patient [3]. The resection success is manually measured by an external neuroradiologist (not part of the study) on a postoperative structural MRI (acquired as part of the clinical routine commonly on the first to third postoperative day). If no residual tumor is detectable, the resection is classified as a gross total resection (binary: yes/no). To aid this assessment, preoperative MRIs can be used for comparison.

#### Secondary endpoints

Health-related quality of life will be measured with the EORTC QLQ-BN20 as an important patient-oriented outcome 7 days and 3 months after surgery. The questionnaire results in a numerical value between 0 and 100, with 100 representing the worst possible score. Therefore, all single-item scores are summed and analyzed using a standard formula as described in the scoring manual [19]. The questionnaire is designed and validated to measure health-related quality of life in brain tumor patients [19].

Functional connectivity in resting-state functional magnetic resonance imaging (fMRI) will be used as a marker for functional reorganization after the intervention (T1) and to give insights into which networks are recruited to compensate for imminent functional loss [20]. Functional connectivity is evaluated in resting-state fMRIs using a seed-to-voxel approach with the seed placed on the stimulated primary motor cortex region. Preprocessing is carried out using fMRIprep [21]. Seed-based connectivity is then quantified as Fisher-transformed correlation coefficients between the motor cortex blood oxygenation level-dependent (BOLD) time series and each individual voxel BOLD time series.

The Action Research Arm Test (ARAT; [22, 23]) is a 19-item observational measure of upper extremity performance. It specifically tests motor performance with respect to four subscales: grasp, grip, pinch, and gross movement. Each task on the ARAT is scored between 0 (patient can perform no part of the test) and 3 (patient performs test normally) [22, 23]. Individual task scores are summed up leading to an overall test score between 0 (worst) and 57 (best score). While the test has not been specifically validated for use in brain tumor patients, it is intensively used in stroke, multiple sclerosis, brain injury, and Parkinson’s disease patients. In these populations, it has proven good reliability and validity and is often used physiotherapists to assess treatment progress.

### Participant timeline {13}

The study is planned to run from March 2023 until July 2025, with recruitment starting in October 2023 in both recruitment centers. Participants are recruited from the regular incoming patients via advertisements, ambulatory care, or the in-patient ward. They will undergo MRI imaging, a clinical assessment, and nTMS assessment as part of the clinical routine. Following these screening procedures, eligible patients will be invited to participate in the study and randomized. The rnTMS intervention will be applied for 5–28 days twice daily, depending on the individually available presurgical time. Each rnTMS session is followed by a short motor training. Ideally on the day following the last rnTMS session, depending on the surgical schedule, patients will undergo resection of the tumor. Follow-up measurements are scheduled immediately after the rnTMS intervention (T1), 7 days postoperatively (T2), and 3 months postoperatively (T3). Table 1 gives an overview of study visits and timepoints as well as the procedures performed per timepoint.

**Table 1:** Participant timeline

**Study procedure**	**Screening**	**Baseline**	**Intervention**	**After intervention**	**Surgery**	**7 days post-operative**	**3 months post-operative**
Visit number		T0		T1		T2	T3
Study day	−1	0	0–5/28	5/28	6/29	13/36	3 months
Informed consent	X						
Inclusion/exclusion criteria	X						
Demographics, medical/neurol. history	X						
nTMS measurement	X			X			
Motor status (BMRC)^a^		X		X		X	X^a^
Structural MRI incl. assessment of GTR^a^	X			X		X^a^	
EORTC-BN20		X		X		X	X
ARAT		X		X		X	X
Diffusion-weighted MRI	X						
Resting-state fMRI	X			X			
rnTMS stimulation			X				
Motor training			X				
(Serious) adverse events^b^		X	X	X	X	X	X
Details of surgery					X		
Tumor histology						X	

Depending on the individually available time before surgery, patients enrolled in the study can have varying treatment durations (5–28 days) and thus, complete their study visits on different study days. The first follow-up measurement (T1) is always performed immediately after the intervention and before surgery. The surgery is then performed on the day after the last treatment, that is, the earliest on study day 6 or the latest on study day 29. Postoperative follow-ups are performed 7 days (T2) and 3 months (T3) after surgery, i.e. the earliest on study day 13 and the latest on study day 36 for T2 and respectively 3 months later for T3

^a^The motor status (BMRC) at T3 and gross total resection rate at T2 are measured as primary endpoints

^b^(Serious) adverse events will be monitored continuously throughout the trial

### Sample size {14}

This is the first study to our knowledge showing the safety and efficacy of rnTMS in glioma patients with regard to postoperative motor status and GTR.

#### Motor status

Data from a small study by Ille et al. (*n* = 19; [11]) demonstrate high effect sizes of 1.66 for the Fugl-Meyer Assessment (FMA) at 3 months follow up in rnTMS compared to sham (mean change in FMA: 31.9 (standard deviation (SD): 18.4) in rnTMS compared to 4.2 (SD: 10.4) in sham, common SD: 16.7). In a meta-analysis combining 10 study estimates for low-frequency rTMS compared to controls in stroke patients, Hsu et al. [24] reported an effect size of 0.69 (95% CI: 0.42–0.95) regarding motor function outcomes. In our study, we aim to demonstrate non-inferiority of motor status at T3 in the rnTMS group compared to sham using the BMRC score (ordinal values between 0 and 5). We use a non-inferiority margin of 0.5 points (difference of group medians), corresponding to the probability of a worse outcome in the intervention group compared to in the control group of *P*(*x* > *y*) = 55.0% (assuming a mean difference of 0.5 and common standard deviation of 2.5, corresponding to an odds ratio (OR) of 1.35 [15], or Cohen’s *d* of 0.17 [16]). We will show that the OR for a worse outcome in the intervention compared to the sham group is significantly below 1.35 (one-sided significance level *α* = 0.025) using 66 sham patients and 66 rnTMS patients. This is possible if the true OR is at least 0.74 (95% CI: 0.23–1.25) (corresponding to a Mann-Whitney measure of P(x > y) = 45.0%, or Cohen’s *d* of − 0.17). This sample size calculation is based on a simplified dichotomous measure, not an ordinal measure, using a similar effect size for an OR of two proportions (50.0% vs. 42,4%, nQuery Advisor 8): When the sample size is 132 (with group 1 and 2 sample sizes of 66 and 66), a one-sided (upper) 97.5% confidence interval for the odds ratio of two proportions will range from the sample proportion odds ratio of 0.74 to an upper limit of 1.24, assuming a worse outcome in 42.4% of patients in the intervention group and in 50.0% of patients in the control group. The confidence interval was calculated using the Simple Asymptotic Method. While we analyze the primary outcome in an ordinal regression model, we expect a similar effect estimate. We demonstrate this at a one-sided significance level of *α* = 0.025 with a power of 80%. Additionally, in case of a 95% CI of the OR for a worse outcome below 1 in the intervention compared to the control we can demonstrate superiority of the intervention.

#### GTR

Until today there are no studies analyzing the GTR in rnTMS compared to sham, but only studies with nTMS. Picht et al. [25] demonstrated a GTR of 61% in the intervention group versus 45% in the control group (OR 1.91). We expect a higher difference in our groups due to the induction of plasticity in this intervention. We expect an OR of 2.85 or higher (for example for proportions of 74% vs. 50% of GTR in the intervention vs. control group). Using information of 66 sham and 66 rnTMS patients it will be possible to demonstrate such a difference using a chi-square test (power=82%, two-sided significance level of 0.05) (nQuery Advisor 8). The statistical analysis will be done using binary logistic regression accounting for the center and age group. Using this analysis strategy will result in at least the same or higher power.

Both primary hypotheses will be tested hierarchically at a two-sided significance level of 0.05. To account for possible dropouts (approximately 10%), 148 patients in total will be included in the study to ensure at least 132 complete cases, even if we apply missing value imputation methods using 30 complete data sets (if missing at random (MAR) or missing completely at random (MCAR) assumption holds). To evaluate if individual missings are MAR, MCAR, or missing not at random (MNAR) we will use information on reasons of missings or dropouts (e.g. if patients drop out due to worsening of health status or not). In case of premature death (before measuring the primary outcome, the BMRC score for those patients will be set to the worst possible value.

### Recruitment {15}

A recent clinical trial [10] applying postoperative rnTMS treatment screened 430 patients with gliomas in the vicinity of motor-relevant areas over a time period of 4 years for study eligibility. In this comparable patient group, 12% of patients did not agree to participate in the study. This would resemble roughly 150 patients willing to participate in the study for each study center across the recruitment period of 19 months, of which 74 have to be recruited.

As part of the study, three different patient workshops will be conducted to involve patients in the study from the beginning. This may have a positive impact on recruitment as patients can give valuable insights on suitable methods and timepoints for study advertisement, while considering the potentially overwhelming preoperative period.

Furthermore, participation in the study will not delay surgery or impact the neurosurgical treatment. We also aim to ease participation in the study by organizing and compensating for travel to the study site if needed.

## Assignment of interventions: allocation

### Sequence generation {16a}

Patients will be randomly allocated (1:1) to the trial arms stratified by recruitment center (Berlin, Heidelberg) and age (< 55 years, ≥ 55 years). The randomization list will be produced by the study biostatistician using a block randomization with varying block sizes.

### Concealment mechanism {16b}

Patients will receive their randomization via the inbuilt tool in the Research Electronic Data Capture (REDCap) software. Different access groups will be defined in the REDCap software, so that only interventional staff has access to the randomization list of patients. Conversely, interventional staff will not have access to document outcomes in the software. These assessments can only be accessed by outcome assessors.

### Implementation {16c}

The randomization list will be used as input for the randomization tool inbuilt in the REDCap software by the trial statistician and recruited patients will be allocated to their respective treatment arms via this software.

Patients are enrolled by a blinded study physician; outcomes are assessed by blinded staff. Only the intervention staff performing the rnTMS intervention are not blinded to the treatment allocation and have access to the randomization list.

## Assignment of interventions: blinding

### Who will be blinded {17a}

The study is designed as a triple-blinded study. Patients, assessors of the outcomes, and the biostatistician are blinded to group allocation of patients. Only the interventional staff performing the rnTMS intervention are not blinded to the treatment allocation and have access to the randomization list. The performing neurosurgeon is informed about the trial participation of the patient but blinded towards the group allocation.

### Procedure for unblinding if needed {17b}

Premature unblinding of a study patient can be considered for medical reasons and in case of severe adverse events. Attending physicians and investigators are encouraged to discuss these requests with the principal investigator. The reason, time, and date of the unblinding will be documented on case report forms (CRF) and study monitors will be informed.

## Data collection and management

### Plans for assessment and collection of outcomes {18a}

The documentation of the surgery as well as the TMS and MRI examination is done via regular patient records. The collection, documentation, and management of experimental data (study data) is done in a study file. Among other things, data on age and gender as well as data on comorbidities and medical history are collected (see {13,19}).

Study data will be collected exclusively in pseudonymous form via digital recording and test forms (eCRFs). These data will be collected and stored via the REDCap platform. Data entry is controlled by access groups ensuring blinding of all personnel. Anatomical/tractography and connectivity data are further stored pseudonymously on a local hospital data server. Electromyographic and navigation data from the TMS examination will be collected via the TMS device and then also stored on the local hospital data server. The name, day, and month of birth of each patient will not be stored in the REDCap database but linked to patient data records by a unique identifier. Corresponding personal data will be maintained in paper format by each participating center. All paper data will be maintained in a locked room and locked cabinet.

### Plans to promote participant retention and complete follow-up {18b}

Regarding the primary outcome measure BMRC score at 3 months follow up we expect a low rate of missings of around 2% [25, 26]. However, to ensure a sufficient number of complete cases the rate of loss to follow up a more conservative 10% rate of dropout is assumed. For the second primary outcome GTR, we expect no missing values since early postoperative structural MRIs are measured as part of the clinical routine and available to all patients.

Postoperative follow-ups are scheduled before release from the ward (T2) or coinciding with regular medical follow-up visits (T3). To promote participant retention, travel to the hospital can be organized by the investigators and financially compensated (for example costs for a taxi). See also {11c}.

### Data management {19}

Study data will be gathered on electronic case report forms, stored in the data management software REDCap, and secured with back-ups of the data dictionary on an external hard drive together with the raw data from TMS and MRI measurements.

Data collection is controlled by an external monitor according to standard operating procedures. The Clinical Trial Office (CTO) at Charité will monitor the correct completion and perform plausibility checks of the data stored in REDCap to avoid discrepancies with the source data. Implausible or missing data will be queried. The data from both study centers will be entered directly in a central REDCap database provided by the coordinating center. Access groups will be defined to assure blinding. REDCap further provides an audit trail for all activities. The coordinating center will provide a training to all users of REDCap during this study.

The database will be locked after completion of data collection and final data review. All relevant study data will be archived by the coordinating investigator for 10 years according to local regulations.

### Confidentiality {27}

The data collected within this study will be used exclusively for the purpose of managing and conducting the study and for the purpose of research and evaluation.

The documentation of all study patients (study data) is done separately from the medical record via a study file. Data collection, data storage, and data analysis will be performed exclusively after pseudonymization. The coding list, which allows the study-related data to be linked to the identifying information of the participants, can only be accessed by the study management and individual persons authorized by it. The coding list will be maintained in paper form. It will be kept in a lockable cabinet and destroyed after ten years. All other documents kept on paper, such as consent forms, etc., will also be kept in a lockable cabinet at the respective study site. Digitally captured pseudonymous study data is stored on the REDCap platform. The study database is also backed up regularly to a local hospital data server. Data collected electronically during the study (anatomical data/tractography and connectivity data/electromyography (EMG) and navigation data from the TMS examination) are also stored pseudonymously on this server. Access to the data collected during the study is regulated within the study team by the principal investigator. The coordinating investigator at the second study center Prof. Dr. Sandro Krieg as well as the members of the study team of the Department of Neurosurgery at Heidelberg University Hospital will be provided with study data for the purpose of evaluation, whereby data transmission will always be in pseudonymized and encrypted form. In the same context, pseudonymized study data of the Heidelberg Center will be made available to the principal investigator Prof. Dr. Thomas Picht, and the staff of the Department of Neurosurgery of the Charité.

The study participants are informed that the study physicians may inspect the patient file for the purpose of evaluation and release the treating physician from the medical confidentiality obligation in this case.

The participants have the possibility to explicitly agree to the transfer of the pseudonymized data to research institutions and clinics within the European Union.

### Plans for collection, laboratory evaluation, and storage of biological specimens for genetic or molecular analysis in this trial/future use {33}

Not applicable, no samples were collected.

## Statistical methods

### Statistical methods for primary and secondary outcomes {20a}

#### Efficacy

To test both main hypotheses of comparable motor status at T3 and a higher GTR in rnTMS patients compared to sham patients, we will apply hierarchical testing at a two-sided significance level of *α* = 0.05 (Power = 80%). Primary hypotheses will be tested in an intention-to-treat (ITT) framework using multiple imputed datasets in case of missing values (under the assumption of MCAR or MAR) after a single imputation of informative missing values (MNAR) for example for patients with worsened health status or patients who die during follow up.

#### Description of the primary efficacy analysis and population

(1) Non-inferiority of motor status at T3 in the rnTMS group vs. sham group will be tested using an ordinal regression model (adjusted for baseline motor status) with dependent variable motor status at T3 (ordinal: six categories). Additionally, center, duration of rnTMS/sham training, and age will be included as covariates. Non-inferiority is successfully proven, if the upper limit of the 95% CI of the odds ratio for a worse outcome in the intervention group is below 1.35 according to the pre-specified non-inferiority margin. (2) The second main hypothesis, that the GTR is higher in the rnTMS compared to the sham group, will be tested (only if the first hypothesis has been proven, hierarchical testing) at a two-sided significance level of *α* = 0.05 using a binary logistic regression model with gross total resection (yes/no) as a dependent variable, group allocation, center, duration of rnTMS/sham training, and age as independent variables. A per-protocol analysis will be conducted as a sensitivity analysis.

#### Safety

The safety analysis includes the calculation of frequencies and incidence rates as well as incidence rate ratios and corresponding 95% confidence intervals of adverse and serious adverse events using Poisson regression or negative binomial regression models depending on the distribution of the variables.

Secondary endpoints: All secondary outcomes will be analyzed using appropriate statistical regression models and by accounting for the particular baseline values (analysis of covariance), center, rnTMS training duration, and age. In the case of measuring endpoints at several time points, we will use mixed regression models to account for the dependencies of the data. A detailed statistical analysis plan will be written and published before the database is closed.

### Interim analyses {21b}

No interim analyses are planned in this study. (S)AEs will be carefully examined regarding their relationship to the intervention by the independent DSMB. In case such a relationship is assumed or cannot be ruled out completely, the following rules apply. Occurrence of any serious or fatal adverse events will lead to the discontinuation of the trial. Occurrence of severe serious and moderate serious adverse events will lead to substantial revisions of the study design, for example, by limiting total treatment duration or reducing the dosage of rnTMS. Actions for not serious severe and moderate adverse events depend on the number of subjects presenting with an adverse event as well as the reversibility of the adverse event. Actions might include similar changes in study design, reductions in dosage, or temporary recruitment stops until further analysis of adverse events. Mild adverse events in any number of subjects as well as moderate not serious (≤ 3 patients if reversible; ≤ 1 patient if not reversible) and severe not serious (≤ 1 patient if reversible) adverse events require no action.

### Methods for additional analyses (e.g. subgroup analyses) {20b}

Differential treatment effects will be analyzed by including interaction terms for subgroups by intervention group. Prespecified subgroups include tumor grade (low-grade glioma vs. high-grade glioma), number of risk factors [13] fulfilled (1 or 2), duration of intervention (sham or rnTMS training, median split), surgeons, and tumor volume. Parallel to interaction effects estimated marginal treatment effects of the subgroups and 95% CI will be reported. Descriptive measures, effect estimates, and 95% confidence intervals will be calculated for each primary and secondary analysis.

Sensitivity analyses include per-protocol analyses and complete case analyses.

### Methods in analysis to handle protocol non-adherence and any statistical methods to handle missing data {20c}

Primary hypotheses will be tested in an ITT framework. The primary population is defined as all randomized patients with a baseline measurement of the primary endpoints. Both primary hypotheses will be tested hierarchically at a two-sided significance level of 0.05. To account for possible dropouts (approximately 10%), 148 patients in total will be included in the study to ensure at least 132 complete cases, even if we apply missing value imputation methods using 30 complete data sets (if MAR or MCAR assumption holds). To evaluate if individual missings are MAR, MCAR, or MNAR we will use information on reasons of missings or dropout (e.g. if patients drop out due to worsening of health status or not). In case of premature death (before measuring the primary outcome), the BMRC score for those patients will be set to the worst possible value.

### Plans to give access to the full protocol, participant-level data, and statistical code {31c}

The study protocol and statistical code for analysis of the primary endpoints will be available on drks.de. Participant-level data will be shared as stated below (see {29}).

## Oversight and monitoring

### Composition of the coordinating center and trial steering committee {5d}

The trial sponsor is the Charité – Universitätsmedizin Berlin with Prof. Thomas Picht, Department of Neurosurgery, as Principal Investigator and Coordinating Investigator. Prof. Sandro Krieg is the leading investigator of the second recruitment site at the Department of Neurosurgery at the University Hospital Heidelberg. PD Dr. Ulrike Grittner, Institute of Biometrics and Clinical Epidemiology, is the study biometrician. The monitoring and auditing will be conducted by the Clinical Study Center, Clinical Trial Office at Charité. An independent external international scientific advisory board will take responsibility as the DSMB.

### Composition of the data monitoring committee, its role and reporting structure {21a}

An independent external Data and Safety Monitoring Board will monitor the trial. The board will consist of two consultant neurosurgeons with experience in nTMS, an expert on biomedical engineering and development of new nTMS technology, and an independent biostatistician. The independent experts will conduct semi-annual meetings starting with recruitment onset to discuss any issues arising from monitoring reports; specifically, the DSMB will evaluate the accumulated trial data for patient safety, study conduct, and progress, and will make recommendations concerning continuation, modification, or termination of the trial.

In case of any unforeseen deaths or occurrence of a serious adverse event (SAE), the board will conduct an ad hoc review of the severity and relatedness of the events to the intervention. Finally, the board will participate in annual meetings organized by the study group. Minutes of these meetings will document any feedback given by the expert committee and be provided to the BMBF/DLR in the interest of full disclosure.

### Adverse event reporting and harms {22}

All (serious) adverse events ((S)AEs) will be collected from the date the informed consent form is signed until the final study visit. For events that occur between postoperative follow-ups, participants are queried on a routine basis about every 2 weeks starting from postoperative day 7. (S)AEs will be captured on case report forms according to MedDRA, including event description; time of onset; assessment of seriousness, severity, relationship to study procedures or interventions, and expectedness; medical care received; outcome of event; and time of resolution/stabilization of the event. All (S)AEs occurring during the trial are documented appropriately regardless of their relationship to the intervention. All (S)AEs will be followed until satisfactory resolution or the participant is stable. All (S)AEs will be assessed by the investigator for severity, expectedness, and relationship to the intervention using the protocol-specified definitions.

Documentation of AEs will be reported to Clinical Trial Management within 7 days. All AEs are reported in aggregate as part of the routine data and safety monitoring report provided to the DSMB and the BMBF/DLR by the Clinical Trial Management. AEs are reported to the IRB as part of the continuing review. SAEs (regardless of expectedness or relatedness) will be reported to the DSMB and BMBF/DLR within 48 h of the investigator becoming aware of the event. Previous trials of the same duration and similar doses did not show any SAEs related to the intervention.

Based on our previous trial on postoperative rnTMS therapy (SAE = 0, AE = 0 in 16 patients with each 7 rnTMS sessions; see in [12]) and pilot study on accelerated rnTMS in healthy subjects (SAE = 0, AE = 5 in 3 subjects with each 14 rnTMS sessions; see in [10]), we expect AEs: *n* = 30 (mild headache); *n* = 50 (tiredness due to intensive stimulation and training); *n* = 10 (dizziness); SAEs: *n* = 0.

### Frequency and plans for auditing trial conduct {23}

During the clinical study, quality control and quality assurance according to the guidelines of the International Council for Harmonisation of Technical Requirements for Pharmaceuticals for Human Use Good Clinical Practice (ICH GCP) will be ensured through monitoring. The CTO at Charité will perform coordination, implementation, and conduction of monitoring at the study sites following its own Standard Operating Procedures. A pre-study visit will be conducted at the participating study site prior to the start of the study, by the coordinating Department of Neurosurgery. Monitoring according to ICH GCP chapter 5.18 including, the monitor will visit the study sites before (initiation visit), during (regular visits), and after completion of the study (close-out visit) to ensure that the study is conducted, recorded, and reported according to the study protocol. Visits on a regular basis during the study are based on risk-based monitoring in accordance with participant enrollment. Key study data will be checked from all patients (e.g., signed informed consent, adherence to inclusion and exclusion criteria, primary endpoint, and safety data (SAEs). All other data are checked based on a representative sample. Additional conference calls with the sites will be performed regularly to maintain adequate quality standards. All (S)AEs (any undesirable experience occurring to a patient during the trial, whether or not considered to be related to the interventional procedure) reported by the patient or observed by the investigators will be reported according to the study protocol, recorded in the case report form, and reported to the neurosurgery department.

### Plans for communicating important protocol amendments to relevant parties (e.g. trial participants, ethical committees) {25}

Important protocol amendments will be communicated to study sites, other relevant parties, and study registries by the project management at Charité. Each study site is responsible for communicating these changes to study patients.

## Dissemination plans {31a}

The results of this study will be published in peer-reviewed journals as open-access and presented at national and international conferences. Study protocols, statistical analysis plans, and results will be published in a public database (drks.de) and as required by regulation. Study findings will further be communicated to the non-scientific audience based on the results of three patient and stakeholder workshops.

## Discussion

The PRECON study is an exploratory phase 2b clinical trial in patients with motor-eloquent brain tumors, aiming to show the efficacy of rnTMS to facilitate the tumor-induced functional reorganization of motor areas. We hypothesize that this reorganization leads to a better or comparable motor function 3 months postoperatively and a higher rate of gross total resections in the intervention group compared to sham stimulation. Based on this exploratory trial it will be possible to get more robust effect estimates for the outcomes and analyze the safety profile of the intervention to proceed to a confirmatory trial. In a larger confirmatory trial, it would also be possible to analyze different secondary endpoints more thoroughly and potential differential treatment effects in subgroups with more power.

Another central aim of this trial is the empowerment of patients. Research shows that participatory research formats can increase transparency and trust in research and therefore is more meaningful and impactful to patients [27]. Our Patient and Public Involvement strategy will also include other stakeholders such as patient advocates, clinicians, scientists, and policymakers. For this purpose, a series of 3 co-creation workshops will focus on different patient-relevant outcomes.

1. Navigating the preoperative phase (month 6): In the first workshop, patients, families, and caregivers will discuss their experiences in the hospital, potential obstacles in navigating through preoperative procedures, and fears associated with this time. A special focus will also be put on patient perspectives on the understanding of illness, disease, therapy interventions, and treatment plans. The participants will develop a concept to guide patients through this initial phase in the clinic. This could be in the form of a brochure or website and will be conceptualized by workshop participants to make it most applicable to them. The final product can then be used to empower further patients in navigating this difficult phase of their disease.
2. Identifying research priorities in neuroplasticity (month 12): In the second workshop, the focus will be placed specifically on understanding of concepts of brain plasticity, neuromodulation, nTMS, and rnTMS as preoperative tools. The aim is to identify new and patient-initiated research topics and questions as well as to discuss and outline study designs, as pragmatic clinical trials. This includes outlining comparators, outcomes and protocols as well as information material and informed consent documents for confirmatory trials.
3. Communicating neuromodulation to the public (month 18): At the end of the project, a final workshop will focus on finding a suitable communication strategy for study results to patients. This involves the development of customized ways of sharing the results of our research in such a way that patients can easily access, understand and consequently further engage in our research and related therapies. The goal of the workshop is a strategy to implement appropriate patient communication to understand the interdependent link between interventions, patient-relevant outcomes (quality of life), and clinical and functional outcomes. This is going to be used to co-create a suitable way to disseminate the results of the study.

## Trial status

Recruitment is planned to start in both study centers in October 2023. The recruitment period is planned to take 21 months.

### Supplementary Information

**Additional file 1.**

## Data Availability

We will share individual participant data after de-identification after publication of the primary analysis in a scientific journal. To this purpose, the consent form for the study will include an opt-in paragraph, where participants can allow the investigators to share their data with interested researchers within the European Union. Interested researchers who provide a methodologically sound proposal should contact the principal investigator by mail. To gain access, data requestors will need to sign a data access agreement.

## Abbreviations

AE: Adverse event
ARAT: Action Research Arm Test
BMBF: Bundesministerium für Bildung und Forschung
BMRC: British Medical Research Council
BOLD: Blood oxygenation level dependent
CRF: Case report form
CTO: Clinical Trial Organization
DLR: Deutsches Zentrum für Luft- und Raumfahrt
DSMB: Data and Safety Monitoring Board
eCRF: Electronic case report form
EMG: Electromyography
EORTC QLQ BN20: European Organization for Research and Treatment of Cancer Quality of Life Questionnaire Brain Tumor Module
FMA: Fugl-Meyer-Assessment
fMRI: Functional magnetic resonance imaging
GTR: Gross total resection rate
ICH GCP: International Council for Harmonisation of Technical Requirements for Pharmaceuticals for Human Use Good Clinical Practice Guidelines
ITT: Intention-to-treat
MAR: Missing at random
MCAR: Missing completely at random
MNAR: Missing not at random
MRI: Magnetic resonance imaging
nTMS: Navigated transcranial magnetic stimulation
OR: Odds ratio
REDCap: Research Electronic Data Capture
rnTMS: Repetitive navigated transcranial magnetic stimulation
SAE: Serious adverse event
SD: Standard deviation
TMS: Transcranial magnetic stimulation
