# Supplementary material for: Preconditioning of the motor network with repetitive navigated transcranial magnetic stimulation (rnTMS) to improve oncological and functional outcome in brain tumor surgery: a study protocol for a randomized, sham-controlled, triple-blind clinical trial
Source: Trials. 2023 Oct 4;24:638. doi: 10.1186/s13063-023-07640-2 (PMC10552254; doi:10.1186/s13063-023-07640-2)
Supplement: Supplementary file 1 — Additional file 1. [file 13063_2023_7640_MOESM1_ESM.zip › EA2_050_19_Votum01032023_German.pdf]

CHARITÉ
UNIVERSITÄTSMEDIZIN BERLIN

Charité | Charitéplatz 1 | 10117 Berlin

Herrn
PD Dr. med. Thomas Picht
Neurochirurgie
CCM

Cc: melina.engelhardt@gmx.de

Ethikkommission
Ethikausschuss am Campus Virchow-Klinikum

Vorsitzende: PD Dr. E. Kaschina
Geschäftsführerin:
Dr. med. Katja Orzechowski

Postadresse
Campus Charité Mitte
Charitéplatz 1 | 10117 Berlin

T +49 30 450 517 222
F +49 30 450 7517 952
ethikkommission@charite.de

Berlin, 01.03.2023

Prekonditionierung motorischer Areale mittels repetitiver navigierter transkranieller Magnetstimulation zur Reduktion des Morbiditätsrisikos in der Hirntumorchirurgie
Antragsnummer: EA2/050/19
Vorgang vom 13.02.2023, Eingang am 17.02.2023, per E-Mail am 28.02.2023

Sehr geehrter Herr Dr. Picht,

hiermit bestätigen wir den Eingang des Schreibens vom 13.02.2023 mit folgenden Anlagen:

- Ethikantrag, Version 3 vom 13.02.2023
- Studieninformation, Version 3 vom 13.02.203
- Einwilligungserklärung, Version 3 vom 13.02.2023

Wir danken für die Kenntnisgabe. Die Ethikkommission erhebt keine Einwände gegenüber den Änderungen.

Mit freundlichen Grüßen

PD Dr. med. Kaschina
Vorsitzende
