# Supplementary material for: Preconditioning of the motor network with repetitive navigated transcranial magnetic stimulation (rnTMS) to improve oncological and functional outcome in brain tumor surgery: a study protocol for a randomized, sham-controlled, triple-blind clinical trial
Source: Trials. 2023 Oct 4;24:638. doi: 10.1186/s13063-023-07640-2 (PMC10552254; doi:10.1186/s13063-023-07640-2)
Supplement: Supplementary file 1 — Additional file 1. [file EA2_050_19_Votum01032023_English.pdf › EA2_050_19_Votum01032023_English.pdf]

Mr.
PD Dr. med. Thomas Picht
Neurochirurgie
CCM

Cc: melina.engelhardt@gmx.de

Preconditioning of the motor network with repetitive navigated transcranial magnetic stimulation (rnTMS) to improve oncological and functional outcome in brain tumor surgery
Application number: EA2/050/19
Docket dated 13.02.2023, received 17.02.2023, emailed 28.02.2023

Dear Dr. Picht,

We hereby acknowledge receipt of the letter dated 13.02.2023 with the following attachments:

- Ethics application, version 3 of 13.02.2023
- Study information, version 3 of 13.02.203
- Informed consent, version 3 of 13.02.2023

Thank you for bringing this to our attention. The Ethics Committee raises no objections to the amendments.

Yours sincerely,

PD Dr. med. Kaschina
Chairwoman
